# Waveform Optimization for the *In Vitro* Detection of Caffeic Acid by Fast-Scan Cyclic Voltammetry

**DOI:** 10.1021/acsmeasuresciau.4c00029

**Published:** 2024-07-31

**Authors:** Joseph N. Tonn, Richard B. Keithley

**Affiliations:** Department of Chemistry, Roanoke College, 221 College Lane, Salem, Virginia 24153, United States

**Keywords:** fast-scan cyclic voltammetry, carbon fiber microelectrode, caffeic acid, flow injection analysis, plant antioxidants

## Abstract

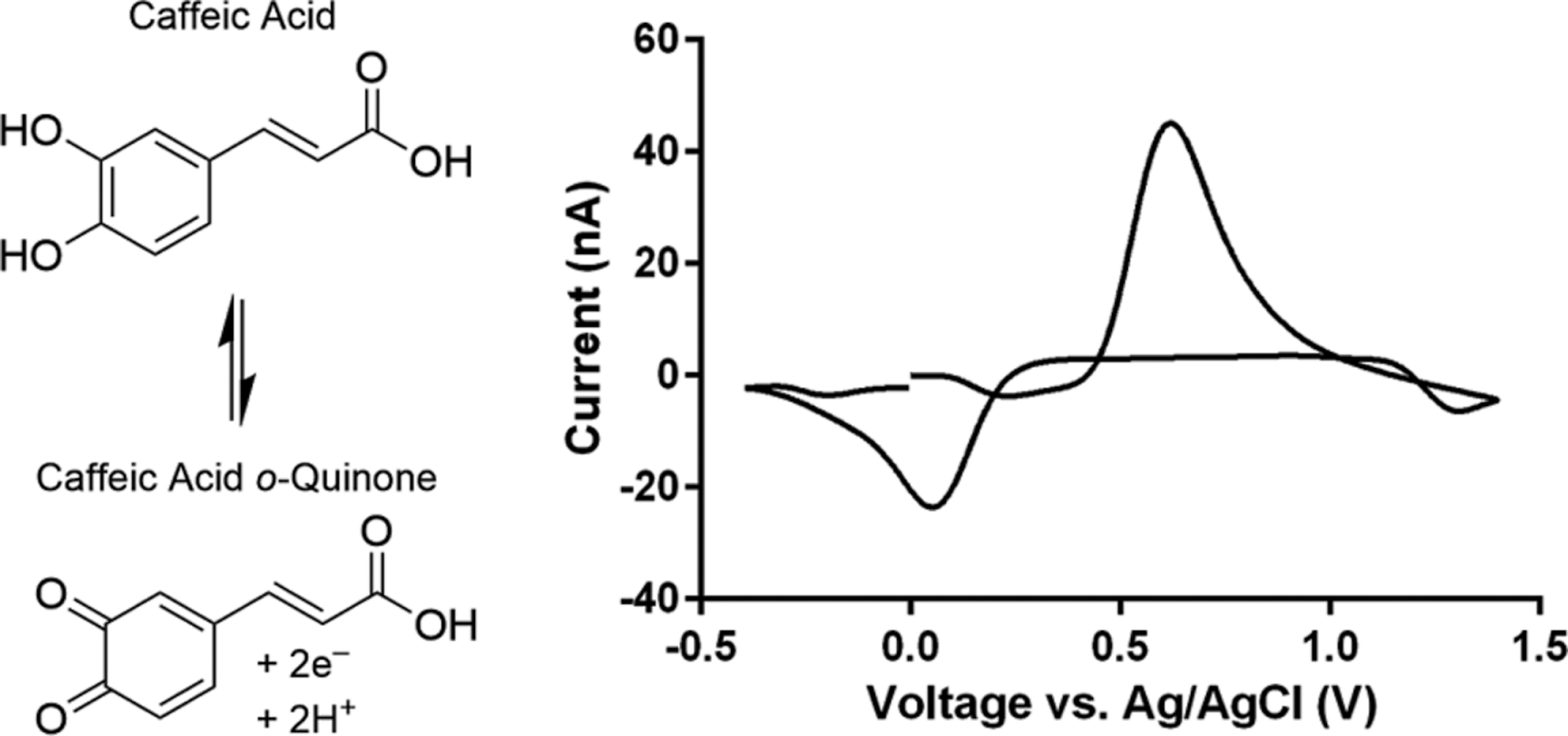

Caffeic acid is a polyphenol of critical importance in plants, involved in a variety of physiological processes including lignin formation, cellular growth, stress response, and external signaling. This small molecule also acts as a powerful antioxidant and thus has therapeutic potential for a variety of health conditions. Traditional methods of detecting caffeic acid lack appropriate temporal resolution to monitor real time concentration changes on a subsecond time scale with nM detection limits. Here we report on the first usage of fast-scan cyclic voltammetry with carbon fiber microelectrodes for the detection of caffeic acid. Through the use of flow injection analysis, the optimal waveform for its detection under acidic conditions at a scan rate of 400 V/s was determined to be sawtooth-shaped, from 0 to 1.4 to −0.4 to 0 V. Signal was linear with concentration up to 1 μM with a sensitivity of 44.8 ± 1.3 nA/μM and a detection limit of 2.3 ± 0.2 nM. The stability of its detection was exceptional, with an average of 0.96% relative standard deviation across 32 consecutive injections. This waveform was also successful in detecting other catechol-based plant antioxidants including 5-chlorogenic acid, oleuropein, rosmarinic acid, chicoric acid, and caffeic acid phenethyl ester. Finally, we show the successful use of fast-scan cyclic voltammetry in monitoring the degradation of caffeic acid by polyphenol oxidase on a subsecond time scale *via* a novel modification of a Ramsson cell. This work demonstrates that fast-scan cyclic voltammetry can be used to successfully monitor real-time dynamic changes in the concentrations of catechol-containing plant polyphenols.

## 1 Introduction

Diverse small molecule phenolics are ubiquitous secondary metabolites within the plant kingdom.^1^ Hydroxycinnamic acids are a major subgroup of these phenolics containing a phenylpropanoid (C_6_–C_3_ benzene-acrylic acid) based structure, with diverse physiological functions.^2−4^ 3,4-Dihydroxycinnamic acid, more commonly known as caffeic acid, is synthesized by all plant species *via* the shikimic/phenylpropanoid pathway and is a major hydroxycinnamic acid in the human diet.^5−7^ Caffeic acid is critical for the formation of lignin, the major component within plant cell walls and the second most abundant biopolymer on Earth (after cellulose).^8,9^ Caffeic acid also serves critical roles in a variety of plant processes, including cellular growth, phototropism, responses to abiotic stressors, and alleopathy, whereby caffeic acid is secreted by plant roots to influence the growth of other nearby species through complex signaling pathways.^10−13^ In addition, caffeic acid acts as a powerful antioxidant.^14−16^ While reactive oxygen species (ROS) serve as essential signaling molecules in plant development, excess ROS can damage cellular tissues so the redox scavenging ability of small molecule antioxidants like caffeic acid is critical for maintaining redox homeostasis in organisms.^17−20^ Caffeic acid has also been shown to demonstrate beneficial pharmacological effects as an antimicrobial compound, a cardioprotective agent, and could have therapeutic benefits for certain cancers, neuronal diseases, and diabetes.^21−24^

**Scheme 1:**
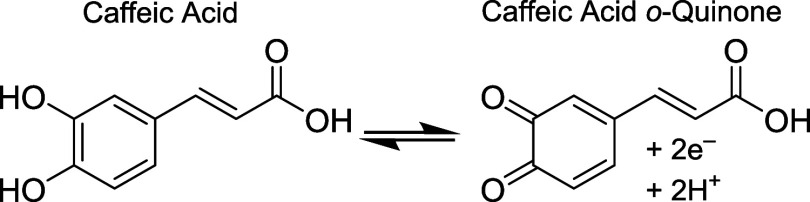
Redox Behavior of Caffeic Acid

Because of its importance, various instrumental methods are available for detecting caffeic acid including ultraviolet–visible (UV–vis), fluorescence, and Raman spectroscopies, chromatography, and electrophoresis.^25−32^ The presence of the catechol moiety within caffeic acid makes it electrochemically active, capable of undergoing a two electron, two proton reduction into caffeic acid o-quinone as shown in Scheme 1.^33^ Indeed, there have been several publications in recent years demonstrating cyclic, square wave, and differential pulse voltammetric approaches capable of measuring caffeic acid, with a focus on chemically modified and nanodecorated macroelectrodes.^32,34−38^ Several recent works have also published comparisons of analytical figures of merit across these various approaches, highlighting nM detection limits or lower available for caffeic acid detection.^32,35,36,39−43^

Fast-scan cyclic voltammetry (FSCV) is an electroanalytical technique that uses voltage sweeps with scan rates above 100 V/s.^44^ An advantage of FSCV over other approaches is that the applied waveform can be applied successively at high frequencies, allowing users to monitor changes in electroactive biomolecules on a subsecond time scale, while maintaining the ability to identify the analyte of interest based on the shapes of the recorded cyclic voltammograms (CV).^44,45^ Coupled with small-sized biocompatible carbon fiber microelectrodes (CFMs), this technique has been used successfully for over 40 years for the real-time detection of neurochemicals in the living brain, primarily catecholamines.^44,46^ More recent FSCV advances have included novel waveforms, CFM modifications for enhanced detection capabilities, and an expansion in the types of analytes detected including amino acids, metal ions, metabolites, peptides, hormones, and other small molecules of biochemical significance.^47−56^

Sordoń et al. have previously shown that CFMs are well-suited for the detection of caffeic acid when used with differential pulse voltammetry and Bavol et al. have used fast-scan differential pulse voltammetry (at scan rates up to 10 V/s) and flow injection analysis (FIA) to detect caffeic acid with a limit of quantitation of 15 μM.^57,58^ The goal of the current work was to develop an approach for the detection of caffeic acid by FSCV at bare CFMs with nanomolar detection limits. Here we report on the optimization of the applied waveform under acidic conditions, the stability of its detection, its interaction with the CFM surface, and the utility of the waveform in monitoring other catechol-based plant phenols. We also demonstrate that FSCV can monitor the degradation of caffeic acid by polyphenol oxidase (PPO) enzyme in real time, using a novel modification of a Ramsson cell^59^ traditionally used for the calibration of CFMs.

## 2 Experimental Section

### 2.1 Reagents

All chemicals were used as received without further purification. Unless otherwise noted, all testing was performed in 0.150 M McIlvaine buffer, prepared daily using sodium phosphate dibasic dodecahydrate (0.100 M) and citric acid monohydrate (0.050 M), both purchased from Thermo Fisher Scientific (Waltham, MA), in 18.2 MΩ ultrapure water produced by a Barnstead EasyPure II system (Thermo Fisher Scientific). pH adjustments were made using hydrochloric acid (VWR International, Radnor, PA) and sodium hydroxide (ThermoFisher Scientific).

Caffeic acid was purchased from TCI America (Portland, OR). 5-Chlorogenic acid was purchased from Chem Impex International (Wood Dale, IL). Oleuropein and chicoric acid were purchased from Cayman Chemical (Ann Arbor, MI). Rosmarinic acid and caffeic acid phenethyl ester were purchased from ThermoFisher Scientific. Stock solutions of these six species, ranging in concentrations between 1–20 mM, were prepared in dimethyl sulfoxide (Thermo Fisher Scientific), stored at −20 °C, and used within 60 days of preparation. Working solutions of each of these species were prepared daily in working buffer as needed. PPO experiments were conducted in a 0.15 M acetate buffer (created by dissolving 0.05 mol sodium acetate trihydrate in a solution of 0.1 mol acetic acid, diluting to volume, and adjusting the pH to 4.25); both chemicals were purchased from ThermoFisher Scientific. PPO (from mushrooms) was purchased from Worthington Biochemical (Lakewood, NJ). PPO stock solutions (∼600 U/mL) were made daily by dissolving an appropriate amount of enzyme into acetate buffer. Active enzyme was kept on ice until use, while denatured PPO was prepared by placing the stock solution into a boiling water bath for 30 min before use.

### 2.2 Electrode Fabrication

CFMs were prepared as previously described.^60^ Individual unsized carbon fibers (grade 34–700, Goodfellow, Huntingdon, U.K.) were first aspirated into glass capillaries (1.2 mm × 0.68 mm × 100 mm, A-M Systems, Sequim, WA) using a vacuum pump (Pittsburgh Automotive, Pittsburgh, CA). Next, capillaries were pulled apart using a micropipette puller (Narishige, Tokyo, Japan). Cylindrical microelectrodes were produced by cutting the exposed carbon fiber to lengths of approximately 75–125 μm using a scalpel and a light microscope (Leica Microsystems, Wetzlar, Germany). An electrical connection was made by backfilling the capillary with 4 M potassium acetate and 0.15 M potassium chloride (ThermoFisher Scientific) using a capillary syringe needle (World Precision Instruments, Sarasota, FL).

### 2.3 FSCV

FSCV experiments were performed in a grounded Faraday cage using a custom system equipped with a Dagan (Minneapolis, MN) potentiostat, a 16-bit PCI-6052E DAC/ADC card (National Instruments, Austin, TX) and Tarheel CV software (version 4.41). Electrodes were preconditioned with the applied waveform for 15 min (10 min at an application frequency of 60 Hz and 5 min at an application frequency of 10 Hz). All data was recorded against a Ag/AgCl reference electrode using the waveform conditions described in the text, with the waveform applied at a frequency of 10 Hz, unless otherwise noted.

FIA was performed using a syringe pump (New Era Pump Systems, Farmingdale, NY) and an electrically actuated six-port valve (Idex Corporation, Lake Forest, IL) controlled with a PCI 6711 card (National Instruments) that switched between buffer, buffer plus analyte, and back to buffer. FIA experiments for waveform optimization were performed using one of two flow conditions. For only those experiments where 10–90% rise time was to be quantified, a flow rate of 2 mL/min was used consistent with other reported FSCV experiments, using an 8 s bolus of analyte. In all other cases, a flow rate of 0.5 mL/min was used with a 30 s bolus of analyte as reported previously to reduce waste.^60,61^ Both conditions provided for peak currents and signal-to-noise ratios (SNRs) that were statistically indistinguishable, but operating the system at a flow rate of 0.5 mL allowed for a substantial reduction in buffer consumption and waste generation during multihour experiments.

Electrode stability experiments included 32 consecutive injections of analyte operated at a flow rate of 0.5 mL/min. Each data file consisted of 10 s of buffer, 30 s of analyte in buffer, and 20 s of buffer, with an additional 50–60 s between each consecutive data file to ensure all analyte was completely flushed out of the flow cell apparatus. Thus, these stability measurements represent data collected over approximately 1.5 h total.

Enzyme experiments were performed in a modified Ramsson cell.^59^ First, an Ag/AgCl reference electrode was placed into a single well of a 24-well cell culture plate (Corning, Corning NY). Next, a CFM was lowered into 995 μL of buffer containing either active or denatured PPO (300 U final concentration), then cycled for 15 min using the optimal waveform. To be clear, enzyme was not electrodeposited onto the electrode surface, but was simply present in solution during the entire test. During each trial, data was recorded for 5 s in the cycling solution, 5 μL of a 200 μM caffeic acid stock solution was then added and rapidly mixed (for a final concentration of 1 μM) and the data was recorded for a total of 50 s to minimize electrode drift.^62^

### 2.4 Data Analysis

All data was filtered using a 2 kHz low pass Bessel filter and digital background subtraction was performed to remove charging current, using an average of ten CVs recorded in the presence of buffer immediately prior to analyte introduction. Current *versus* time traces were obtained at the oxidation potential of the analyte and SNRs were calculated by dividing the maximum current value by the standard deviation of the baseline noise generated over five seconds prior to analyte introduction.

All numerical values are reported as the average ± standard error of the mean. For all experiments except the enzyme work, the response of every individual electrode was calculated from the average of three trials, meaning data are replicates (N) of triplicates. Enzyme data N refers to single electrodes tested with and without active enzyme. All statistical analyses were performed using GraphPad Prism (version 6 for Windows, GraphPad Software, San Diego, CA).

## 3 Results and Discussion

### 3.1 Buffer Conditions and Initial Testing

*In vitro* FSCV detection of neurotransmitters traditionally uses HEPES, TRIS, PBS, or artificial cerebrospinal fluid-based buffers, pH adjusted to 7.4 to mimic the *in vivo* environment. *In vitro* detection of caffeic acid, however, typically occurs under acidic solutions^36,39,57,63^ as caffeic acid can rapidly decompose in solutions above pH 5.^64^ For this work, McIlvaine buffer^65^ (phosphate/citrate) was used because of its good buffering capacity over a wide range of acidic pH values and it has been previously shown to be compatible with FSCV measurements under moderate applied voltage conditions.^66,67^

**Figure 1:**
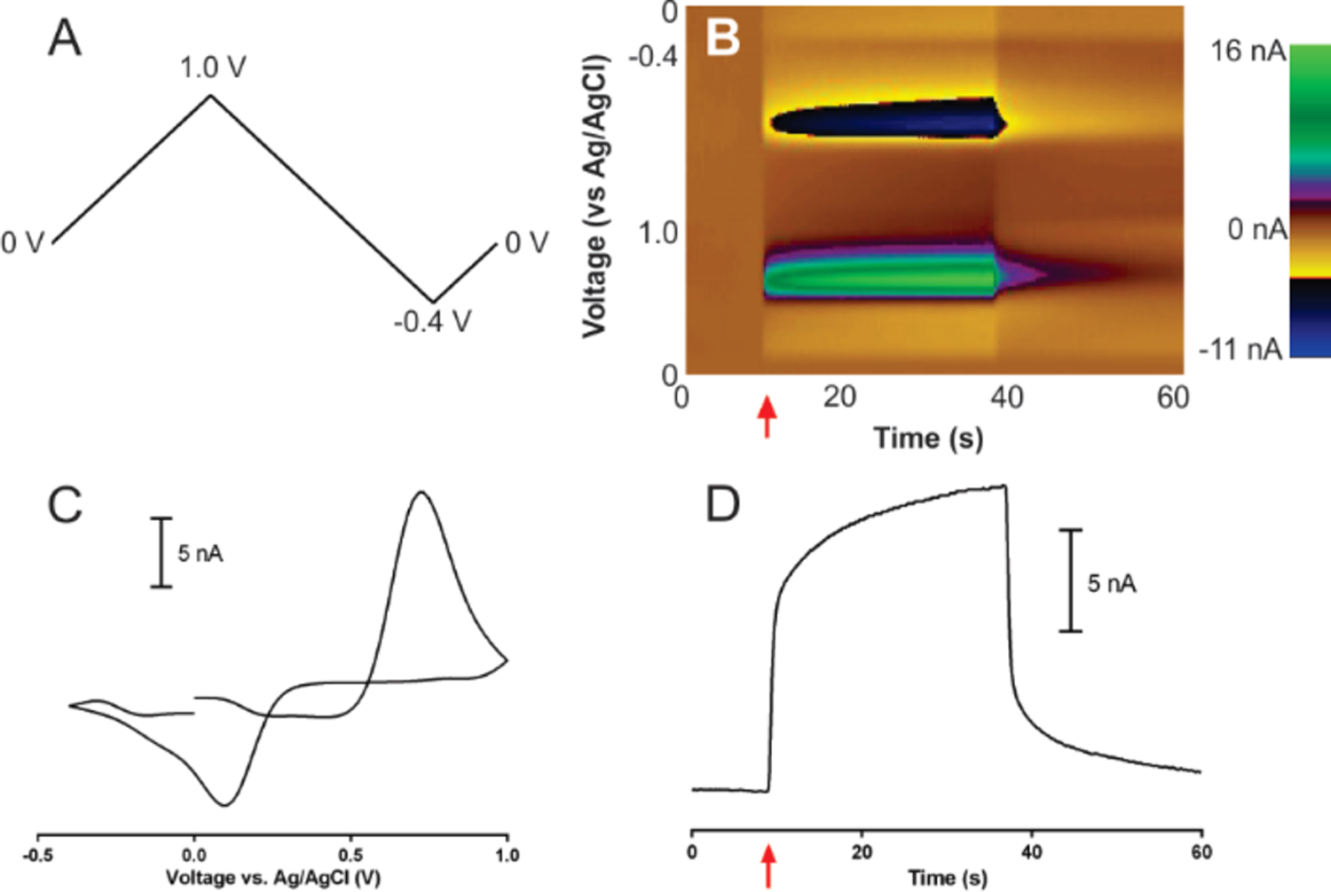
FSCV detection of 1 μM caffeic acid *via* FIA (McIlvaine buffer, pH 3). Panels (A–D) show the waveform used at 400 V/s, the resulting color plot, the CV obtained at the end of the analyte bolus and the current *versus* time trace obtained at the maximum oxidation potential. The red arrows in (B, D) indicate the start of the analyte bolus.

Initial testing of caffeic acid by FSCV utilized pH 3 McIlvaine buffer and a sawtooth-shaped waveform at 400 V/s as shown in Figure 1A. A holding potential of 0 V was chosen because at this pH, ∼95% of caffeic acid in solution exists in the neutral protonated form as its p*K*_a_ is approximately 4.4.^33^ The anodic limit, the cathodic limit, and the scan rate were chosen as 1.0, −0.4 V, and 400 V/s, respectively, as these limits have been shown to provide for good detection of catechols at CFMs.^68^ Because of the high scan rate, a large charging current is produced in FSCV that is digitally subtracted out using a recorded signal in buffer prior to the introduction of analyte during FIA measurements.^44^ The resulting background-subtracted FIA measurement of a representative 1 μM caffeic acid injection is shown as a color plot in Figure 1B. Each vertical slice of the color plot represents a CV recorded at one instance in time, each horizontal slice represents a current *versus* time trace at a specific potential, and current is recorded in false color.

The background-subtracted CV of caffeic acid recorded at the end of the bolus is shown in Figure 1C. Quasi-reversible electron transfer is apparent, with an oxidation voltage at approximately 0.7 V on the forward sweep and a reduction peak at approximately 0.1 V on the reverse sweep. There was also a small negative peak in the CV between 0.2 and 0.5 V on the forward sweep. This peak has been shown previously in FSCV-FIA measurements^44,49,69^ and is non-Faradaic in origin, caused by changes in capacitance due to analyte adsorption onto the CFM surface.^70−72^ The current *versus* time trace at the oxidation potential of caffeic acid (Figure 1D) shows the expected behavior of an FIA experiment: a sharp increase in current approaching steady-state conditions, followed by a sharp decrease in signal as the analyte bolus is removed.^60,61^

### 3.2 Waveform Optimization

**Figure 2:**
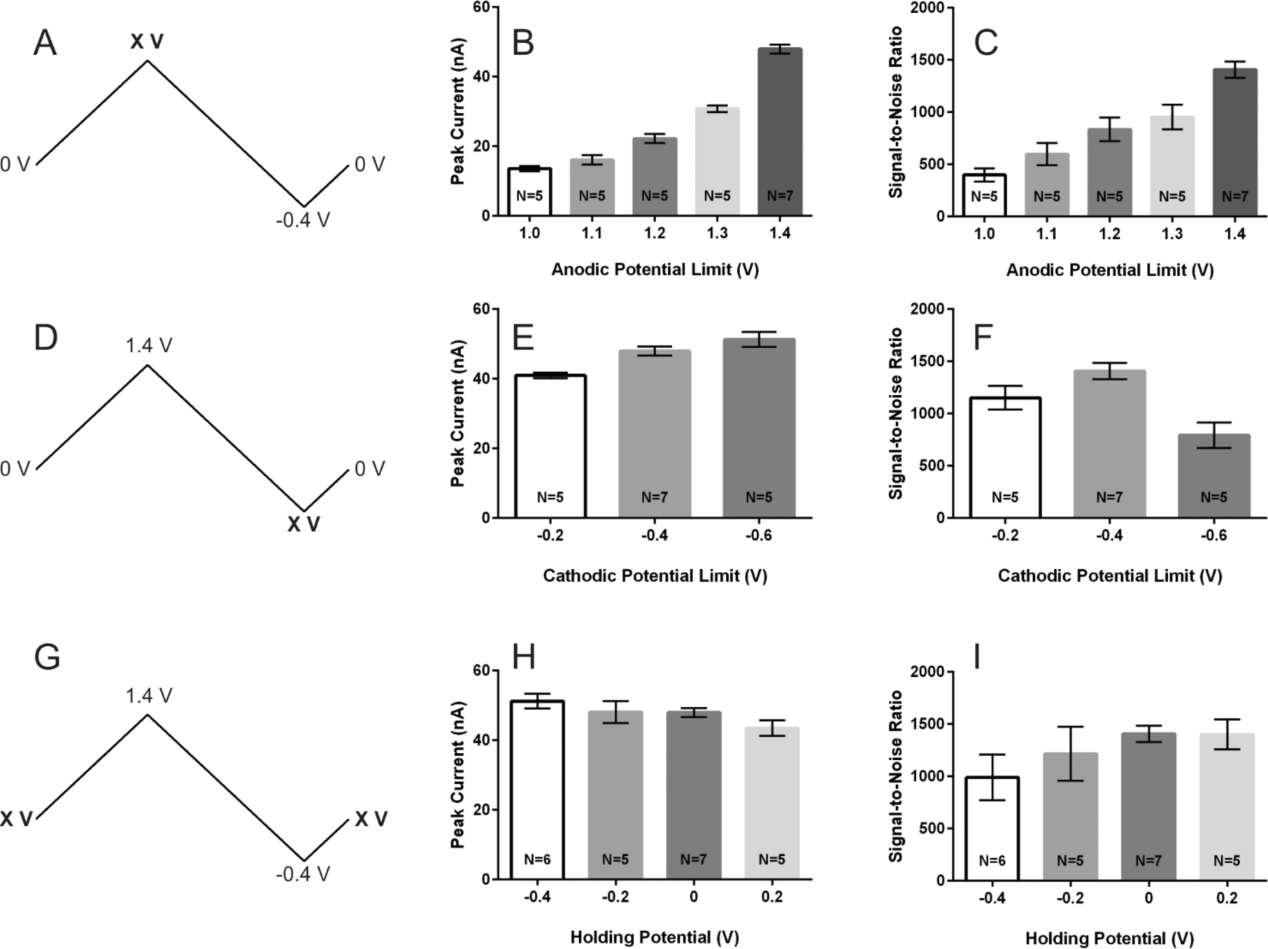
Assessing FSCV peak currents (second column) and FIA SNRs (third column) obtained from FIA experiments of 1 μM caffeic acid as a function of waveform (first column). The top row represents alteration of the anodic potential limit, the middle row represents the alteration of the cathodic potential limit, and the bottom row represents the alteration of the holding potential, each denoted as the letter X in the waveform drawing within the corresponding row. All recordings were performed in McIlvaine buffer, pH of 3.

After initial testing demonstrated caffeic acid was able to be successfully detected by FSCV, the potential limits of the waveform were modified iteratively to maximize sensitivity and minimize detection limit. Two metrics were quantified for the assessment within FIA current *versus* time traces, peak current and SNR, as altering potential limits can influence both sensitivity and CFM noise levels.^68,73^ Increasing the anodic potential limit as shown in Figure 2A–C demonstrated a significant change in both sensitivity (*P* < 0.001) and SNR (*P* < 0.001), with an anodic potential limit of 1.4 V providing both the highest peak current and SNR for caffeic acid detection (see Tables S1 and S2 in Supporting Information). This result supports previous work as increasing the potential limit is known to electrochemically oxidize the surface of CFMs, enhancing sensitivity toward catechols by creating more oxygen-containing functional groups for enhanced adsorption.^44,68,73,74^ Anodic potential limits higher than 1.4 were not tested due to solvent breakdown.^44^

The cathodic potential limit was changed next, while maintaining the anodic potential limit of 1.4 V previously determined (Figure 2D–F). Altering the cathodic potential limit did significantly influence peak current (ANOVA, *P* = 0.0013) and SNR (ANOVA, *P* = 0.0025). A voltage of −0.4 V was determined to provide the best balance between peak current and signal-to-noise ratio (see Tables S3 and S4 in Supporting Information), with the −0.6 V limit significantly increasing noise level of the CFM for caffeic acid detection. Changing potential limits on FSCV waveforms can alter surface morphology and produce highly reactive radical species, either of which may have contributed to the increase in noise seen at the most negative potential limit.^68,75^

Finally, the holding potential was changed while maintaining the anodic potential limit of 1.4 V and the cathodic potential limit of −0.4 V (Figure 2G–I). The holding potential did not significantly affect peak current (ANOVA, *P* = 0.143) or SNR (ANOVA, *P* = 0.306). Altering the interscan holding potential in FSCV significantly affects the measured signal for charged species that preconcentrate onto the CFM surface,^44^ but as the vast majority of caffeic acid in solution is neutral at the pH of 3, it was expected that altering the holding potential would not significantly influence sensitivity or SNR. While altering the holding potential did not provide for a statistically significant change in SNR, the average SNR did decrease and the electrode-to-electrode variability increased as the holding potential was made more negative as shown in Figure 2I. In FSCV measurements, a CFM is operated at its holding potential nearly 90% of the time, with this value influencing CFM stability and electrode fouling.^75^ The increase in electrode-to-electrode variability shown here with a more negative holding potential mirrors a similar result reported by Heien when optimizing the cathodic holding potential limit in a triangular-shaped waveform used for dopamine detection.^73^

Increasing the holding potential did cause the non-Faradaic capacitive dip present in the CV during the forward scan to move more positive, approaching the oxidation peak of caffeic acid. This effect is consistent with earlier work,^72^ so maintaining a holding potential of 0 V also minimizes any confounding of caffeic acid quantitation whereby this dip could be conflated with the oxidation peak. Thus, the optimal waveform was determined to be 0 to 1.4 to −0.4 to 0 V at the scan rate tested here, 400 V/s. However, as our waveform was optimized under acidic conditions and the performance of CFMs are known to vary with pH,^69,76^ any future work investigating caffeic acid within an *in vivo* environment may require additional waveform optimization beyond the work completed herein.

### 3.3 pH Influence

**Figure 3:**
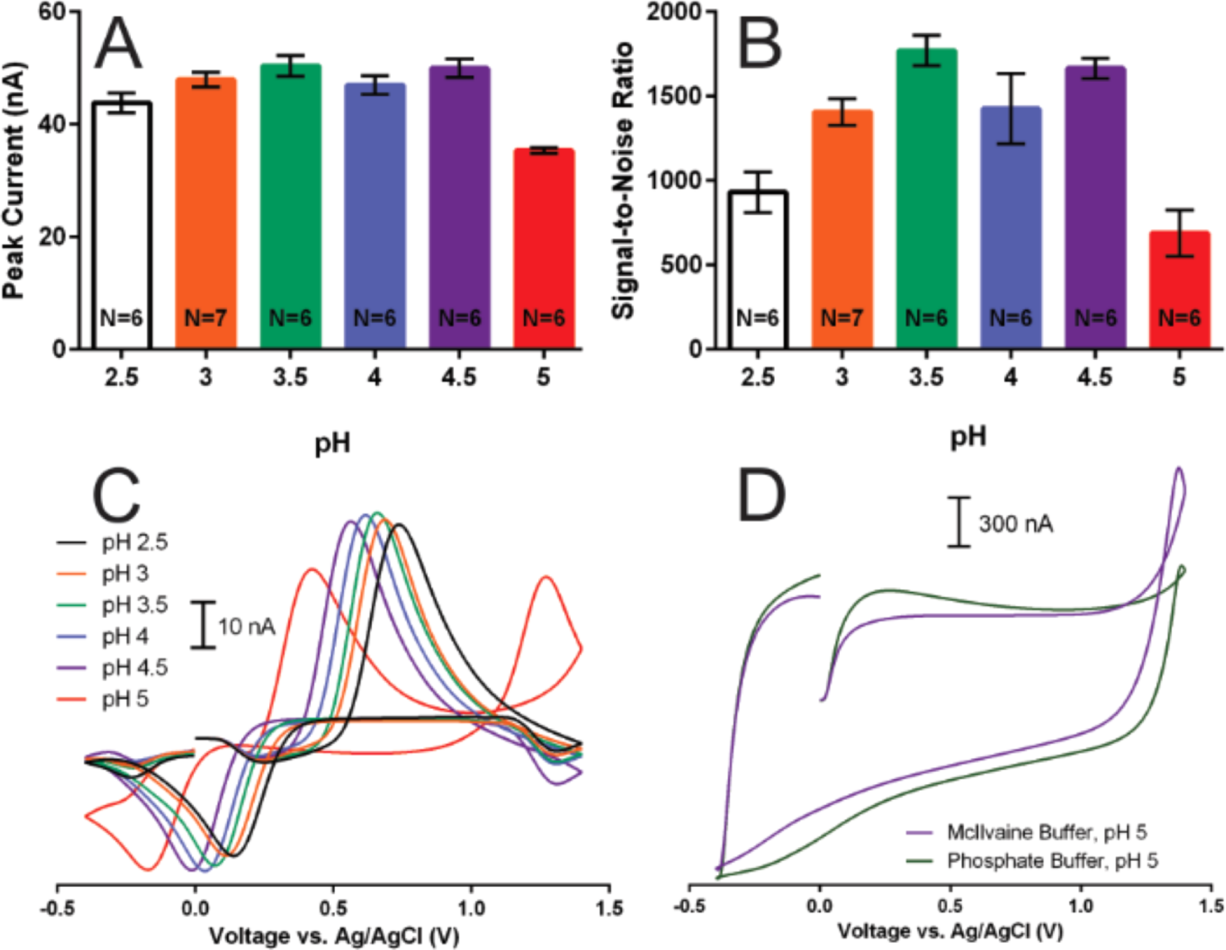
pH effects on the detection of 1 μM caffeic acid in McIlvaine buffer. Peak currents and SNRs obtained from current *versus* time traces obtained *via* flow injection analysis experiments are shown in panels (A, B), respectively. Panel (C) shows representative CVs of 1 μM caffeic acid as a function of buffer pH. Panel (D) shows background currents of the same CFM in McIlvaine buffer (0.100 M phosphate, 0.050 M citrate) and phosphate buffer (0.100 M), both of pH 5.

Once the optimum waveform was determined, the influence of pH on the measured caffeic acid signal was investigated, with the results shown in Figure 3. pH values between 2.5 and 5 were studied, corresponding to the lower limit of the buffering range of the solvent up to a workable *in vitro* stability limit of caffeic acid in solution.^64,65^ Specifically, Cilliers and Singleton have reported that caffeic acid in solution can undergo autoxidative degradation whose extent varies with pH and temperature. They reported that over a 6 h period, at pH values of 4, 6, and 8, caffeic acid can degrade less than 1%, approximately 7%, and up to 76%, respectively.^64,77^ This pH range is also consistent other *in vitro* electrochemically based caffeic acid assays as stated above,^36,39,57,63^ is appropriate for extractions of caffeic acid from plant tissue,^78^ and is consistent with the extracellular xylem fluid of some plants.^79^

pH significantly influenced caffeic acid peak current (*P* < 0.0001) and SNR (*P* < 0.001) over the range tested as shown in Figure 3A,B. There were no significant differences in peak currents or SNRs for caffeic acid detection by FSCV across pH values of 3, 3.5, 4, and 4.5 (see Tables S5 and S6 in Supporting Information). While caffeic acid peak current at pH 2.5 was not significantly different from those recorded at these other pH values, its SNR was lower due to an increase in noise. This result may indicate issues with CFM sensor stability under such acidic conditions.

Caffeic acid showed significantly lower peak currents at pH 5 compared to other pH values tested and showed significantly lower SNRs compared to those recorded across pH values of 3, 3.5, 4, and 4.5 as shown in Tables S5 and S6 in Supporting Information, respectively. Figure 3C shows representative CVs for 1 μM caffeic acid at each of the pH values tested. As the pH of the solution increased, there was a consistent negative shift in peak potentials, as expected for a redox process involving proton transfer.^33,63,69^ However, at a pH of 5, there was a much more dramatic negative shift in peak potentials, and a second peak present near the anodic switching potential limit. The CFM surface is known to undergo changes with pH, and pH 5 has previously been shown to be an inflection point when assessing trends in the Faradaic component of FSCV charging current that is associated with the redox of functional groups on the CFM surface.^76^

We further investigated this effect by examining the FSCV background current, as shown in Figure 3D. In McIlvaine buffer at pH 5, a large unstable hysteresis was present (Figure 3D, purple trace). Thus, the peak recorded near the switching potential in the caffeic acid CV at pH 5 in Figure 3C was not due to caffeic acid electrochemistry, but likely due to errors in digital background subtraction, a result seen previously for other analytes.^80^ If the buffer was reformulated to contain only phosphate by removing citrate, the hysteresis present in the charging current was eliminated (Figure 3D, green trace) indicating citrate is the cause of this hysteresis. However, it should be noted that phosphate alone has poor buffering capacity at pH 5 and cannot be used for analytical testing. Previous reports of McIlvaine buffer usage in FSCV have not reported this artifact associated with citrate, but the applied voltages used in those works never increased above 1.2.^66,67^ Overall, these results demonstrate that caffeic acid detection in McIlvaine buffer is optimal across pH values of 3–4.5, though the usage of noncitrate buffers may allow for testing at pH 5 or above with the waveform developed here. As stated previously, our work focuses on *in vitro* caffeic acid studies under acidic conditions, an environment that is not consistent with *in vivo* mammalian studies that operate at a pH of 7.4. Additional pH studies would be warranted under these circumstances for any potential future work in this area.

### 3.4 Calibration, Stability, and Scan Rate Studies

**Figure 4:**
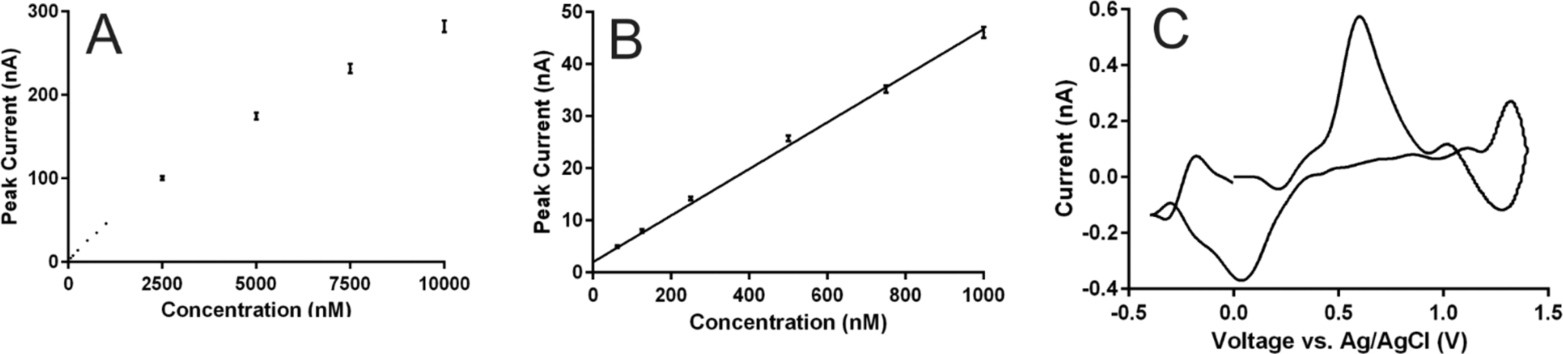
Caffeic acid dynamic range studies. Panel (A) represents peak current recorded up to concentrations of 10 μM (*N* = 5). Panel (B) represents the linear region of the calibration curve, with an *R*^2^ of 0.996. Panel (C) shows a representative cyclic voltammogram recorded at a concentration of 12.5 nM caffeic acid. All measurements were recorded in McIlvaine buffer, pH 4.

After the waveform was optimized and its performance assessed across a wide series of pH values, the dynamic range of FSCV caffeic acid detection was determined, as shown in Figure 4. Peak current increased with concentration up to the highest value tested (10 μM, Figure 4A), though the limit of linearity of the calibration curve was 1 μM (Figure 4B). Sensitivity was determined to be 44.8 ± 1.3 nA/μM and the limit of detection (LOD) was determined to be 2.3 ± 0.2 nM. A representative CV of caffeic acid at 12.5 nM is shown in Figure 4C. While its SNR is low, as expected when operating near the LOD, this result shows that FSCV is successfully able to monitor caffeic acid at nM concentrations.

**Figure 5:**
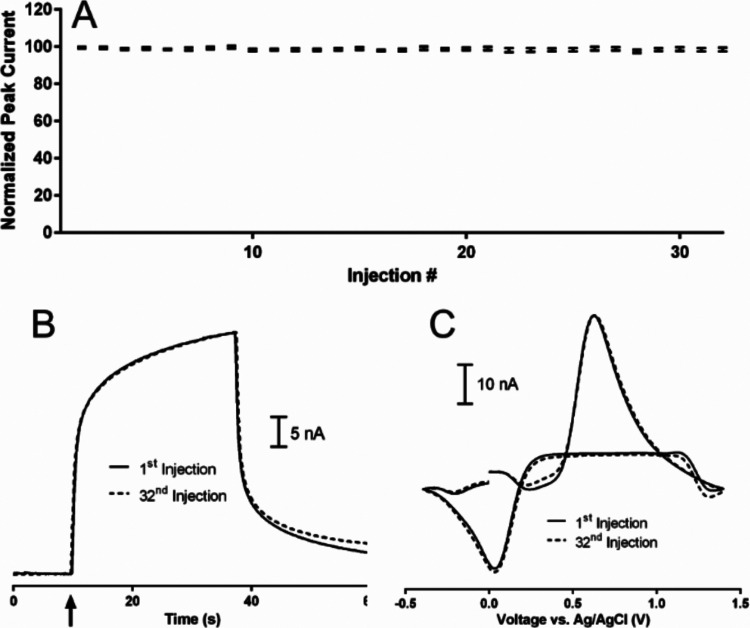
Caffeic acid stability studies. Panel (A) represents peak current, normalized to the first injection, recorded for thirty-two consecutive injections of 750 nM caffeic acid (*N* = 5). Panels (B, C) show overlays of the current *versus* time traces recorded at the oxidation potential and the CVs from the first and thirty-second injection, respectively. The arrow in panel (B) represents the onset of the caffeic acid bolus. All measurements were recorded in McIlvaine buffer, pH 4.

After dynamic range testing, stability testing was performed, in a manner consistent with other reports,^50,81,82^ with the results shown in Figure 5. Panel 5A shows peak currents, normalized to the first injection, for 32 consecutive FIA measurements of 750 nM caffeic acid, recorded over the course of approximately 1.5 h. There was no significant difference in peak current across the 32 injections (ANOVA *P* = 0.165, *N* = 5), and the average relative standard deviation in peak currents across all electrodes was 0.96%. Figure 5B,C show overlays of the first and thirty-second current *versus* time traces and the cyclic voltammograms, respectively. There was virtually no difference in signal recorded, demonstrating the stability of caffeic acid detection by FSCV is excellent.

**Figure 6:**
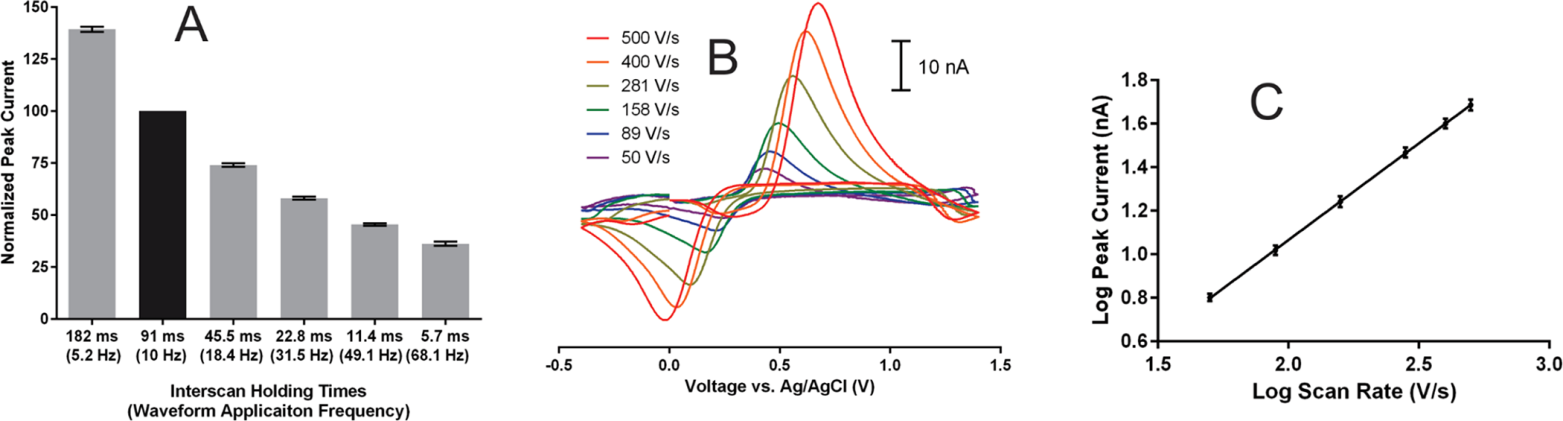
Adsorption studies. Panel (A) represents peak current for 750 nM caffeic acid FIA injections with different holding times between successive waveform applications at 400 V/s, normalized to that recorded with a holding time of 91 ms (10 Hz application frequency, black bar), *N* = 5. Panel (B) shows representative 750 nM caffeic acid CVs recorded at various scan rates on the same electrode at a waveform application frequency of 10 Hz. Panel (C) shows a log–log peak current *versus* scan rate plot for the data shown in (B), for *N* = 5 electrodes. The *R*^2^ of the line of best fit was 1.000. All measurements were recorded in McIlvaine buffer, pH 4.

Additional experiments were performed to assess how caffeic acid interacts with the CFM surface during FSCV measurements by changing the waveform application frequency at a single scan rate of 400 V/s (Figure 6A) and changing scan rate at a single waveform application frequency of 10 Hz (Figure 6B,C). As the waveform application frequency increased, decreasing the amount of time between successive scans, peak current significantly decreased (ANOVA, *P* < 0.0001). When plotting the log of peak current *versus* the log of scan rate, theory predicts species with purely diffusion-mediated transport would have a slope of 0.5, while those with purely adsorption-mediated transport would have a slope of 1.0.^83^ The log–log plot of caffeic acid peak current *versus* scan rate in Figure 6C with the optimized waveform was linear with a slope of 0.89.

Both sets of data demonstrate caffeic acid strongly adsorbs onto the CFM surface, even with a 0 V holding potential. Based on the chemical structure of caffeic acid shown in Scheme 1, the mechanisms for this interaction likely involve hydrogen bonding with surface-contained oxygen functional groups, and pi-pi stacking onto CFM basal planes. Surface-contained carboxyl groups have been reported to have a p*K*_a_ of approximately 4.8 and 5.2,^84^ only some of which would be deprotonated under the acidic conditions tested here, though these may be occupied by counterions from buffer salts at much high concentration than analyte. Roberts previously identified the importance of carbonyls and hydroxyls in the adsorption of the catecholamine dopamine onto oxidized carbon electrodes,^74^ which may also hold true for caffeic acid in our work.

### 3.5 Detecting Other Catechol-Based Plant Polyphenols with the Optimized Waveform

**Figure 7:**
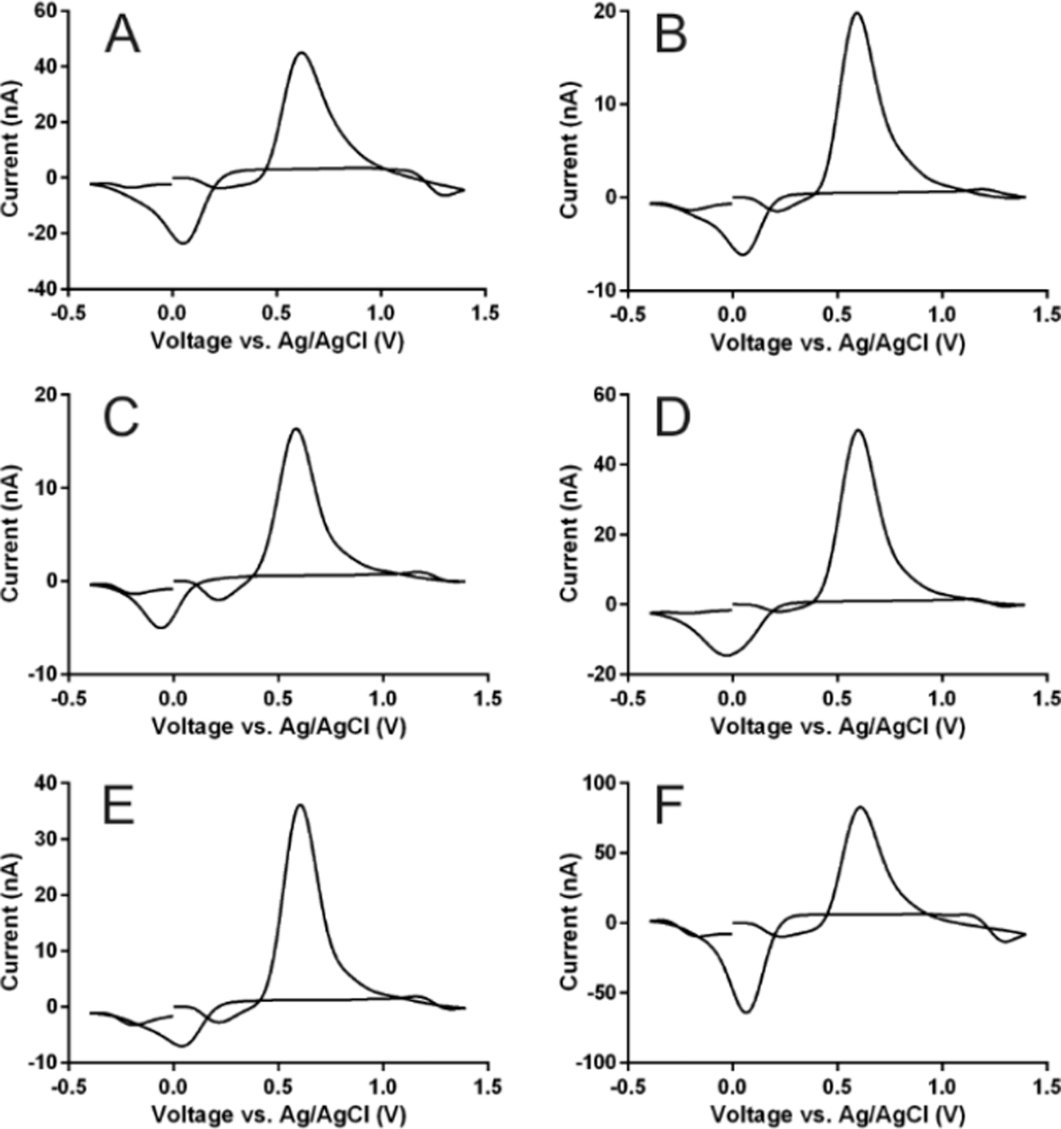
Representative CVs for caffeic acid (A), 5-chlorogenic acid (B), oleuropein (C), rosmarinic acid (D), chicoric acid (E), and caffeic acid phenethyl ester (F) using the optimized FSCV waveform. The concentration of all species was 1 μM, and all recordings were performed in McIlvaine buffer, pH 4.

To test the broader applicability of the waveform developed here, other electroactive catechol-containing plant polyphenols were studied by FSCV including 5-chlorogenic acid, oleuropein, rosmarinic acid, chicoric acid, and caffeic acid phenethyl ester.^85−89^ Chemical structures for these species are shown in Figure S1 (Supporting Information) and representative CVs for their detection using the caffeic acid waveform are shown in Figure 7. Analytical figure of merits for their detection are shown in Table 1, including sensitivity (m), LOD, limit of quantitation (LOQ), 10–90% rise time (*t*_R_) during an FIA injection, oxidation peak potential (*V*_P,OX_), reduction peak potential (*V*_P,RED_), and peak separation (Δ*E*_P_).

**Table 1:** Analytical Figures of Merit for the Detection of Caffeic Acid and Selected Derivatives by FSCV Using the Optimized Waveform in McIlvaine Buffer, pH 4^a^

	caffeic acid	5-chlorogenic acid	oleuropein	rosmarinic acid	chicoric acid	caffeic acid phenethyl ester
*m* (nA/μM)	44.8 ± 1.3	19.4 ± 0.6	16.4 ± 0.7	49.9 ± 1.2	36.1 ± 2.1	82.5 ± 3.9
LOD (nM)	2.3 ± 0.2	5.3 ± 0.2	5.3 ± 0.3	1.9 ± 0.1	2.4 ± 0.3	1.4 ± 0.2
LOQ (nM)	7.6 ± 0.5	17.8 ± 0.6	17.7 ± 0.9	6.4 ± 0.4	8.1 ± 0.9	4.5 ± 0.7
10–90% *t*_R_ (s)	2.35 ± 0.03	0.95 ± 0.10	0.88 ± 0.05	1.46 ± 0.08	1.09 ± 0.04	2.69 ± 0.06
*V*_p,OX_ (V)	0.615 ± 0.002	0.595 ± 0.008	0.590 ± 0.002	0.597 ± 0.008	0.597 ± 0.008	0.612 ± 0.001
*V*_p,RED_ (V)	0.050 ± 0.001	0.040 ± 0.006	–0.070 ± 0.002	–0.030 ± 0.005	0.030 ± 0.005	0.064 ± 0.001
Δ*E*_P_ (mV)	565 ± 2	556 ± 2	660 ± 4	627 ± 3	567 ± 4	548 ± 2

a *N* = 5 for each Analyte. Abbreviations are defined in the text.

As all species contain a catechol group, the electrochemical characteristics are very similar. All compounds displayed quasi-reversible electron transfer, had oxidation potentials near 0.6 V, and had reduction potentials near 0 V. However, there were significant differences in sensitivity and time response across these species. Except for rosmarinic acid, there was a general trend between increased sensitivity and increased response time among the analytes, suggesting that their interactions with the CFM surface are also heavily adsorption-dependent. Caffeic acid phenethyl ester had the highest sensitivity (nearly double that of caffeic acid) while oleuropein had the lowest (nearly one-third the sensitivity of caffeic acid). Caffeic acid phenethyl ester contains a phenyl ring within its structure while oleuropein contains a glucose moiety, which suggests analyte adsorption *via* pi–pi stacking onto the basal planes of the graphitic structure of the CFM surface can significantly enhance detection of these polyphenols.

These results demonstrate that while the optimized waveform is robust enough to detect several different plant polyphenols, selectivity would be a challenge in systems where more than one species exists. This effect is already known within FSCV detection of catecholamine neurotransmitters, as dopamine and norepinephrine, which differ by only a hydroxyl group on the molecular backbone, yield virtually identical CVs.^90−92^ FSCV is still an appropriate technique for their detection, provided users take appropriate precautions when designing their experiments.^90−92^ These precautions, as outlined by Phillips and Wightman, include anatomical/physiological verification, pharmacological verification, and characterization by independent chemical methods.^92^ These approaches have previously been used to facilitate FSCV separately characterizing dopamine and norepinephrine in real systems despite their identical voltammetric shapes,^90,91^ overcoming the selectivity concern and providing promise for the detection of catechol-containing plant phenols with similar challenges. Novel data processing strategies already suggested by the literature could also allow for distinguishing among these catechol-containing plant polyphenol species in the future as well. FSCV has a multidecade history of successfully utilizing various chemometric approaches to separate overlapping signals for example.^90,93−95^ Additionally, Schmidt and McElligott^96^ recently highlighted supervised machine learning techniques such as those used by Moran et al.^97^ that successfully separated serotonin and dopamine. Choi et al. also recently reported on the success of deep learning, a strategy used in artificial intelligence, to separate highly overlapping CVs with reduced error over more traditional chemometric approaches.^98^ These tools, used in tandem with the widely applicable waveform developed here and the precautions outlined by Phillips and Wightman, should allow for improved selectivity in systems where multiple catechol-containing plant polyphenols coexist.

### 3.6 Monitoring Caffeic Acid Degradation by PPO

**Figure 8:**
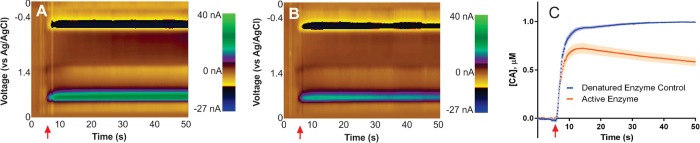
Caffeic acid degradation by PPO using a modified Ramsson cell. Panel (A) shows a color plot for a control recording of a caffeic acid injection (1 μM final concentration) into a solution of denatured PPO (300 U final concentration). Panel (B) repeats the experiment on the same electrode in a solution of active enzyme. Panel (C) shows average responses (solid line) with error (shading) from *N* = 13 total electrodes. The red arrows indicate the introduction of caffeic acid into the buffer solution containing enzyme (denatured or active).

Finally, an experiment was performed to demonstrate the utility of FSCV in monitoring changes in caffeic acid concentrations on a subsecond time scale. PPO is an enzyme that is known to oxidatively degrade caffeic acid primarily into its quinone form, which itself is highly reactive and can rapidly degrade into multiple condensation products, in addition to other oligomeric/polymeric products, as thoroughly described by Cheynier and Moutounet.^99^ FIA was insufficient for performing this experiment as the time associated with preparing the solutions and performing a recording is longer than the reaction between caffeic acid and PPO under normal conditions. Instead, we modified a Ramsson cell, previously developed for CFM calibration,^59^ into a system appropriate for rapid real-time reaction monitoring. In this setup, the CFM is lowered into a Petri dish containing enzyme and cycled with the applied waveform to activate the CFM surface and prepare its recording. Once activated, the signal is recorded for several seconds before a small volume of caffeic acid is added and mixed rapidly in solution using a micropipette. The reaction is then allowed to proceed for the rest of the recording. The results of the PPO experiment within the modified Ramsson cell are shown in Figure 8.

For each electrode, two trials were performed: CFMs were cycled once in a solution of denatured enzyme as a control and once in a solution of active enzyme. Representative color plot recordings from these trials are shown in Figure 8A,B, with an average across all electrodes tested (*N* = 13) shown in Figure 8C. In the control reaction containing denatured enzyme in solution, caffeic acid introduction resulted in rapid steady-state conditions, as expected if the denatured enzyme was not capable of reacting with it. However, in the trial containing active enzyme, caffeic acid introduction resulted in a lower peak concentration and a continual decrease in its concentration with time, over the remaining ∼45 s of recording. This experiment successfully demonstrated the ability of FSCV to monitor changes in caffeic acid concentration on a subsecond time scale. Moreover, this work demonstrates that a Ramsson cell is a simple, yet powerful tool that can act as a chemical reactor vessel appropriate for monitoring *in vitro* chemical reactions by FSCV.

## 4 Conclusions

Overall, we demonstrate that FSCV is a successful approach for monitoring caffeic acid and other plant-based catechol-containing polyphenols with nanomolar detection limits. A sawtooth-shaped waveform was developed that facilitated detection in solution conditions appropriate for maintaining their stability from pH 3–4.5. The novel modification of the Ramsson cell also allowed for real-time monitoring of changes in caffeic acid concentrations, demonstrating a significant advantage of FSCV over other electrochemical methods. Beyond enzymatic reactions, this work facilitates the subsecond monitoring of ROS neutralization by plant antioxidants and allows for the study of signaling dynamics of catechol-containing polyphenols in plant systems. Our approach could also be used for the analysis of plant extracts containing these critical species as well. However, because the CFM surface (and thus analyte sensitivity) varies with pH, additional waveform optimization may be needed prior to detection of these species within mammalian *in vivo* systems, as our waveform was developed and optimized under more acidic conditions.

## Supporting Information Available

Complete list of all *P*-values for all ANOVA multiple comparison post-testing within Figures 2 and 3, along with structures for analytes tested in Figure 7 (PDF)

## Abbreviations

CFM: carbon fiber microelectrode
CV: cyclic voltammogram
FSCV: fast-scan cyclic voltammetry
PPO: polyphenol oxidase
SNR: signal-to-noise ratio
