# Supplementary material for: Waveform Optimization for the In Vitro Detection of Caffeic Acid by Fast-Scan Cyclic Voltammetry
Source: ACS Meas Sci Au. 2024 Jul 31;4(5):534–45. doi: 10.1021/acsmeasuresciau.4c00029 (PMC11487675; doi:10.1021/acsmeasuresciau.4c00029)
Supplement: Supplementary file 1 — tg4c00029_si_001.pdf [file tg4c00029_si_001.pdf]

Supporting Information

Waveform Optimization for the *In Vitro* Detection of Caffeic Acid by Fast-Scan Cyclic Voltammetry

Joseph N. Tonn and Richard B. Keithley*

Roanoke College, Department of Chemistry, 221 College Lane, Salem, VA 24153, USA
* e-mail: keithley@roanoke.edu

Table S1. Tukey's multiple comparisons test results of the data shown in Figure 2B, comparing 1 µM caffeic acid peak currents as a function of the anodic potential limit.

Anodic Potential Limit (V)	Significant?	Significance Level	P-value
1.0 vs. 1.1	No	N/A	0.617
1.0 vs. 1.2	Yes	***	0.0006
1.0 vs. 1.3	Yes	****	< 0.0001
1.0 vs. 1.4	Yes	****	< 0.0001
1.1 vs. 1.2	Yes	*	0.0171
1.1 vs. 1.3	Yes	****	< 0.0001
1.1 vs. 1.4	Yes	****	< 0.0001
1.2 vs. 1.3	Yes	***	0.0006
1.2 vs. 1.4	Yes	****	< 0.0001
1.3 vs. 1.4	Yes	****	< 0.0001

Table S2. Tukey's multiple comparisons test results of the data shown in Figure 2C, comparing 1 µM caffeic acid signal-to-noise ratios as a function of the anodic potential limit.

Anodic Potential Limit (V)	Significant?	Significance Level	P-value
1.0 vs. 1.1	No	N/A	0.632
1.0 vs. 1.2	Yes	*	0.0403
1.0 vs. 1.3	Yes	**	0.0061
1.0 vs. 1.4	Yes	****	< 0.0001
1.1 vs. 1.2	No	N/A	0.474
1.1 vs. 1.3	No	N/A	0.126
1.1 vs. 1.4	Yes	****	< 0.0001
1.2 vs. 1.3	No	N/A	0.915
1.2 vs. 1.4	Yes	**	0.0021
1.3 vs. 1.4	Yes	*	0.0169

Table S3. Tukey's multiple comparisons test results of the data shown in Figure 2E, comparing 1 µM caffeic acid peak currents as a function of the cathodic potential limit.

Cathodic Potential Limit (V)	Significant?	Significance Level	P-value
-0.2 vs. -0.4	Yes	*	0.0119
-0.2 vs. -0.6	Yes	**	0.0011
-0.4 vs. -0.6	No	N/A	0.275

Table S4. Tukey's multiple comparisons test results of the data shown in Figure 3F, comparing 1 µM caffeic acid signal-to-noise ratios as a function of the cathodic potential limit.

Cathodic Potential Limit (V)	Significant?	Significance Level	P-value
-0.2 vs. -0.4	No	N/A	0.199
-0.2 vs. -0.6	No	N/A	0.0805
-0.4 vs. -0.6	Yes	**	0.0018

Table S5. Tukey's multiple comparisons test results of the data shown in Figure 3A, comparing 1 µM caffeic acid peak currents as a function of buffer pH.

pH	Significant?	Significance Level	P-value
2.5 vs. 3	No	N/A	0.377
2.5 vs. 3.5	No	N/A	0.0505
2.5 vs. 4	No	N/A	0.684
2.5 vs. 4.5	No	N/A	0.0780
2.5 vs. 5	Yes	**	0.0059
3 vs. 3.5	No	N/A	0.849
3 vs. 4	No	N/A	0.997
3 vs. 4.5	No	N/A	0.926
3 vs. 5	Yes	****	< 0.0001
3.5 vs. 4	No	N/A	0.630
3.5 vs. 4.5	No	N/A	> 0.9999
3.5 vs. 5	Yes	****	< 0.0001
4 vs. 4.5	No	N/A	0.748
4 vs. 5	Yes	***	0.0001
4.5 vs. 5	Yes	****	< 0.0001

Table S6. Tukey's multiple comparisons test results of the data shown in Figure 3B, comparing 1 µM caffeic acid signal-to-noise ratios as a function of buffer pH.

pH	Significant?	Significance Level	P-value
2.5 vs. 3	No	N/A	0.0859
2.5 vs. 3.5	Yes	***	0.0006
2.5 vs. 4	No	N/A	0.0847
2.5 vs. 4.5	Yes	**	0.0030
2.5 vs. 5	No	N/A	0.736
3 vs. 3.5	No	N/A	0.292
3 vs. 4	No	N/A	> 0.9999
3 vs. 4.5	No	N/A	0.656
3 vs. 5	Yes	**	0.0024
3.5 vs. 4	No	N/A	0.388
3.5 vs. 4.5	No	N/A	0.9900
3.5 vs. 5	Yes	****	< 0.0001
4 vs. 4.5	No	N/A	0.753
4 vs. 5	Yes	**	0.0028
4.5 vs. 5	Yes	****	< 0.0001

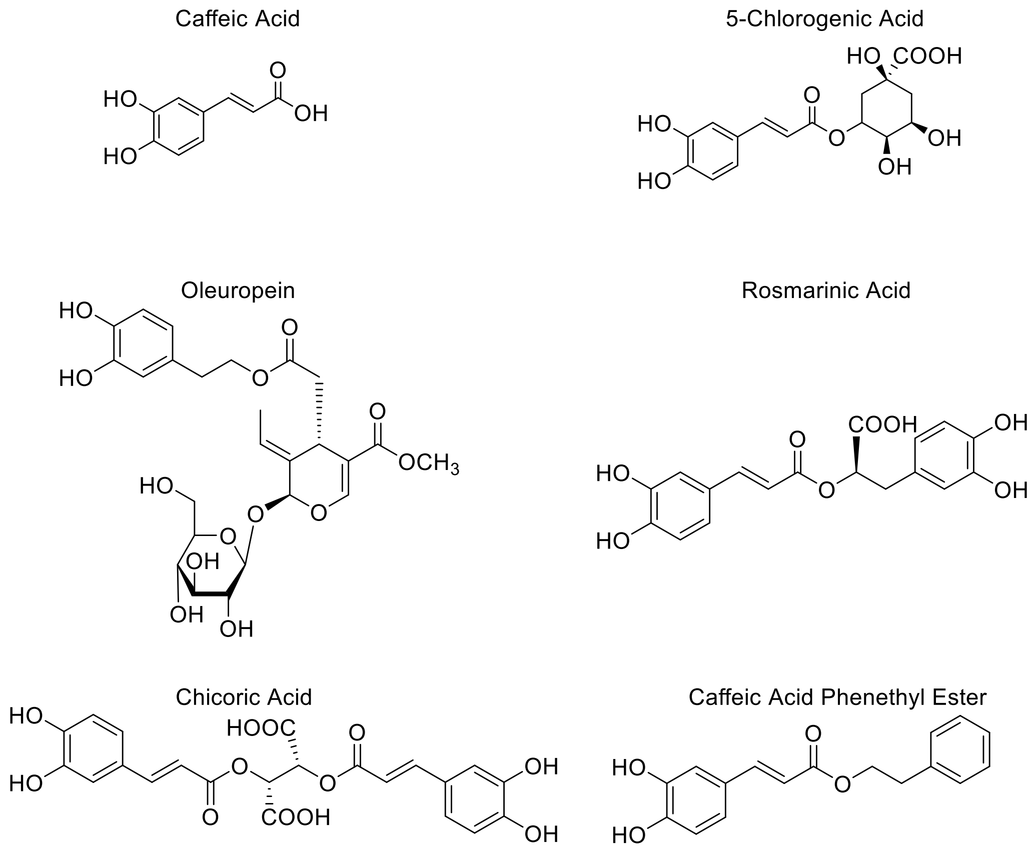

Figure S1. Chemical structures of analytes tested in Figure 7.
